# Comparison of Herbal Agents with Sodium Hypochlorite as Root Canal Irrigant: A Systematic Review of *In Vitro* Studies

**DOI:** 10.1155/2021/8967219

**Published:** 2021-11-25

**Authors:** Kavalipurapu Venkata Teja, Krishnamachari Janani, Kumar Chandan Srivastava, Deepti Shrivastava, Jerry Jose, Anand Marya, Mohmed Isaqali Karobari

**Affiliations:** ^1^Department of Conservative Dentistry & Endodontics, Saveetha Dental College & Hospitals, Saveetha Institute of Medical & Technical Sciences, Saveetha University, Chennai, Tamil Nadu 600077, India; ^2^Department of Conservative Dentistry and Endodontics, SRM Dental College, SRM Institute of Science and Technology, Ramapuram, Chennai-89, India; ^3^Oral Medicine & Radiology, Department of Oral & Maxillofacial Surgery & Diagnostic Sciences, College of Dentistry, Jouf University, Sakaka 72345, Saudi Arabia; ^4^Periodontics, Department of Preventive Dentistry, College of Dentistry, Jouf University, Sakaka 72345, Saudi Arabia; ^5^Department of Orthodontics, Faculty of Dentistry, University of Puthisastra, Phnom Penh 12211, Cambodia; ^6^Department of Restorative Dentistry & Endodontics, Faculty of Dentistry, University of Puthisastra, Phnom Penh 12211, Cambodia

## Abstract

During endodontic treatment, eliminating microorganisms from the root canals should be considered with utmost importance. Before filling the canal, every effort should be made to ensure optimal shaping and adequate disinfection of the root canal system. This systematic review aimed to compare the efficacy of herbal agents with sodium hypochlorite (NaOCl) in reducing the microbial load while used as a root canal irrigant. The research question in the present study was to assess “Is there a significant difference in reducing microbial load comparing sodium hypochlorite (NaOCl) and herbal agents.” Electronic databases (PubMed, Scopus, and Web of Science) were searched from their start dates to November 2020 using strict inclusion and exclusion criteria and reviewed following PRISMA (Preferred Reporting Items for Systematic Reviews and Meta-Analyses) guidelines. Only *in vitro* studies comparing herbal agents with NaOCl regarding antimicrobial efficiency were included. Two reviewers independently assessed the included article. 825 articles were obtained from an electronic database. Twenty papers were included for review of the full text. Eleven papers were excluded because they did not meet the inclusion criteria. Finally, nine articles were included in the systematic review. The present systematic review was at the *in vitro* level; therefore, the result cannot translate the exact clinical conditions. This systematic review concludes that herbal agents cannot be used as a main irrigant for canal disinfection.

## 1. Introduction

The root canal of infected teeth is usually polymicrobial, existing as a biofilm. The ideal outcome of an endodontic treatment should target the elimination of microorganisms and their byproducts established within the root canal system [1]. Cases with persistent infection where the microorganisms are not completely eliminated need more attention as in the majority of cases it leads to reinfection [2]. To attain this, mechanical preparation of the root canal alone may not be sufficient. A disinfection regimen should emphasize optimal shaping such that the disinfectant reaches inaccessible areas such as anastomosis, isthmus, and the lateral canal [3].

An ideal irrigant should possess antimicrobial properties, an ability to dissolve the remnant necrotic pulp tissue, and should cause minimal irritation to the periapical tissue [4]. Different concentrations of sodium hypochlorite (NaOCl) are commonly used as root canal irrigants as they possess the majority of the properties mentioned above for an ideal root canal irrigant. Its uniqueness among available root canal irrigants is that it can dissolve the necrotic and the remnant vital pulp tissue [4, 5]. However, it irritates the periapical tissues when extruded beyond the apex, eventually leading to pain during root canal treatment.

In order to avoid complications associated with the usage of NaOCl, many authors have suggested the usage of herbal agents as an alternative or as an adjunct to the conventional root canal irrigants [6, 7]. Several herbal agents such as essential oil, triphala, green tea polyphenol, *Morinda citrifolia*, neem, tulsi, German chamomile, orange peel extract, and essential oregano oil have been studied to assess the antibacterial properties against the most resistant endodontic and periodontal pathogen [8–10]. Therefore, this systematic review was undertaken to investigate the effect of herbal irrigants in comparison with NaOCl.

## 2. Materials and Methods

### 2.1. Rationale of Systematic Review

This systematic review aimed to assess the antimicrobial efficiency of herbal agents with sodium hypochlorite. This systematic review was conducted following the PRISMA 2020 (Preferred Reporting Items for Systematic Reviews and Meta-Analyses).

### 2.2. Objectives

The present systematic review included articles published on the conventional agent (NaOCl) and herbal agents. The search was performed using multiple terms till November 2020.

### 2.3. PICOS Question

The research question was constructed based on the following:

  Population: extracted human teeth infected with *Enterococcus faecalis* and *Candida albicans*
  Intervention: all herbal irrigants
  Comparison: 1–5.25% sodium hypochlorite (NaOCl)
  Outcome: assessment of microbial reduction
  Studies: *in vitro* studies
  Principle: do the herbal irrigants differ in antimicrobial efficiency compared to NaOCl

### 2.4. Search Strategy

**Figure 1:**
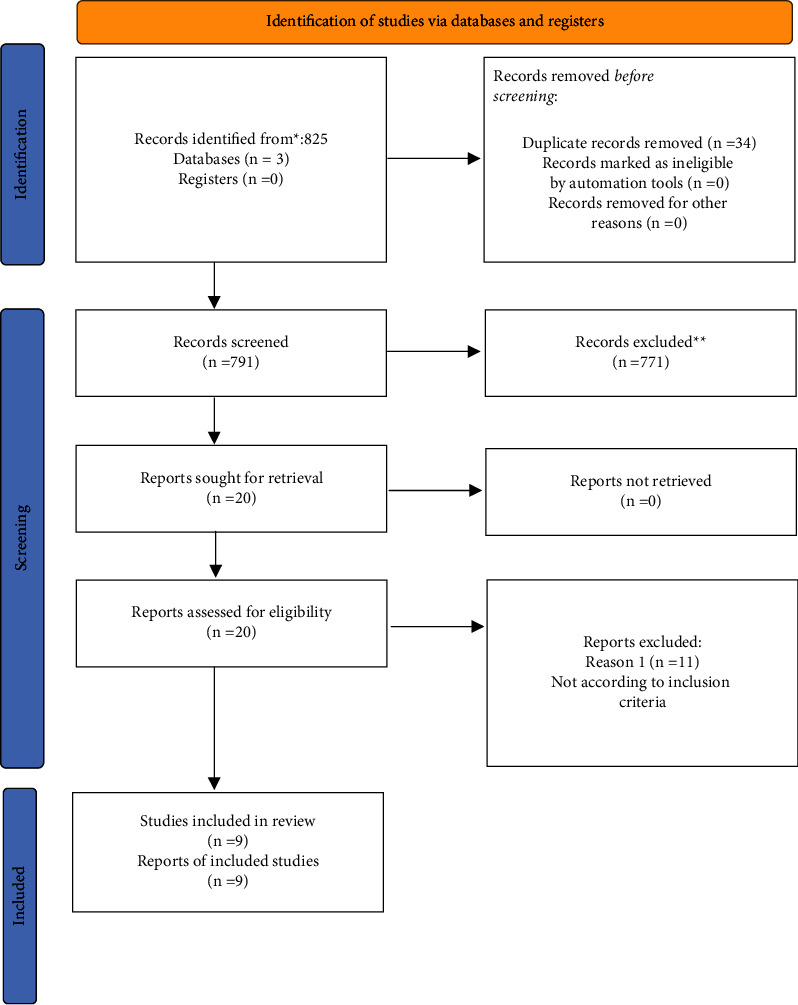
PRISMA flow diagram. ^*∗*^ Consider, if feasible to do so, reporting the number of records identified from each database or register searched (rather than the total number across all databases/registers). ^*∗∗*^ If automation tools were used, indicate how many records were excluded by a human and how many were excluded by automation tools.

Search terms related to root canal dentin, endodontic treatment, irrigants, herbal agents, and antimicrobial efficiency were searched for potential articles until November 2020. The databases used for the search were PubMed, Scopus, and Web of Science. The search strategy was modified based on the database used. A representative search strategy (used for PubMed) has been shown in Figure 1.

### 2.5. Eligibility Criteria

  Type of studies:
  (i) *In vitro* studies and (ii) root canal irrigation performed on extracted teeth.

#### 2.5.1. Inclusion Criteria

The herbal agents were compared with NaOCl for antimicrobial efficiency while performing an *in vitro* root canal treatment.

Full-text articles in English were selected.

The search was performed from different databases and duplicates were removed. Based on the eligibility criteria, the title and abstract of the article were carefully appraised to include the articles that matched the scope of the systematic review. Full-text articles were assessed for further analysis. Two independent reviewers performed the analysis as abovementioned and in the situation of any discrepancies, it was sought by the third reviewer.

#### 2.5.2. Exclusion Criteria

  Animal studies and review articles
  Full-text articles in languages other than English were excluded.

### 2.6. Data Extraction

Two independent reviewers performed the data extraction from the full text article included in the review. The outcome measure of this review compared the antimicrobial efficiency of herbal irrigants with NaOCl. Variables such as sample size, choice of irrigants, volume and concentration of irrigant, choice of the needle, and the method of sample collection were assessed.

## 3. Results

A total of 825 articles were obtained from an electronic database, out of which 34 were duplicate records and hence were removed. Out of 791 articles, 771 articles were excluded following the title search. Twenty papers were included for the full-text review. Another eleven papers were excluded because they did not meet the inclusion criteria. Finally, nine articles were included in the systematic review (Table 1). A summary of article selection is presented as a flowchart, based on PRISMA guidelines (Figure 1). The general characteristics of the included article were tabulated separately for antimicrobial efficiency (Table 2).

### 3.1. Selection of Teeth and Sample Size

All the studies included for the review have chosen only single-rooted teeth for analysis with a sample size ranging from of 40 to 180 (Table 2).

The risk of bias was high in all the papers for blinding and randomization as these details were not mentioned. All papers had a low risk of bias in data reporting; four out of nine studies reported low risk for standardization of protocol. The overall risk of bias was considered high.

### 3.2. Antimicrobial Efficiency (Tables 3 and 4)

#### 3.2.1. Size of Apical Preparation

Four studies have performed the root canal preparation using a K file [11, 12, 17, 18]. In the study performed by Rosaline et al. [14], the size of root canal preparation was not mentioned. Sharifian et al. [16], in their study, used low-speed round bur for canal preparation, whereas a rotary file system was used for the preparation of root canals in the remaining studies [5, 13, 15].

#### 3.2.2. Microbial Inoculation

Out of the nine studies included in the review, two studies did not mention the strain of *Enterococcus faecalis* used for their study [11, 18]. Two studies did not mention the adjusted suspension of colony forming units (CFU) of the strain [16, 17].

#### 3.2.3. Irrigation Protocol

Five studies did not mention the protocol followed for root canal irrigation [5, 11, 16–18]. The remaining four studies mentioned the irrigation protocol which they followed in their studies [12–15].

#### 3.2.4. Volume of Irrigant and Time of Irrigation

The other important aspect for a successful outcome of disinfection is the volume of the irrigant used and its time of contact within the root canal system. Out of included studies, two studies have not mentioned the volume of the irrigant used [14, 16, 17]. Concerning the contact time of the irrigant, no details were mentioned in the study conducted by Divia et al. 2018 [17].

#### 3.2.5. Choice of Irrigation Needle and Irrigant Activation

The choice of needle used for root canal disinfection has a considerable impact on the penetration depth of the irrigant, irrigant flow pattern, and shear stress exerted on the canal wall. Five studies have not mentioned the gauge of the needle and tip design [5, 11, 14, 16, 17]. Two studies have performed irrigation with a 30-gauge needle [12, 15]. Sedigh-Shams et al. [13] used a 27-gauge needle, whereas Arvind Kumar et al. [18] used a 25-gauge needle. However, the remaining studies have not mentioned the choice of the needle. Among the included articles, none of the studies has mentioned the agitation device used for disinfection.

#### 3.2.6. Sample Collection

Most of the included articles used sterile paper points to collect the samples from the canal. Out of all the studies, only one study has not mentioned the sample collection method [17]. In the study by Kumar et al. [18], an H file was used to collect the sample from the root canal.

#### 3.2.7. Culture Method

Rosaline et al. [14] used confocal laser scanning microscopy to evaluate the remaining bacterial adhesion to root dentin. On the other hand, Gupta–Wadhwa et al. [15] and Kumar et al. [18] used polymerase chain reaction (PCR). Remaining studies performed CFU's assessment to assess antimicrobial efficiency. The assessment of antimicrobial activity is mentioned in Table 5.

### 3.3. Risk of Bias

Cochrane criteria for risk of bias were modified according to *in vitro* studies by evaluating the domain about present reviews such as randomization, standardized operator protocol, blinding, and data reporting. Blinding in these studies implies blinding of the evaluator. The risk of bias was scored as low when the details of the parameters as mentioned earlier were mentioned with no ambiguity, but when there was ambiguity, they were scored as unclear. When no details were mentioned, it was scored as high.

Cochrane criteria were modified by taking into consideration a few parameters for the quality assessment [19]. For the antimicrobial studies, all the included articles reported about the type and number of canals. None of the studies mentioned whether a single operator performed all the experimental procedures. Four studies reported a high risk of bias as they did not mention the volume of the irrigant used, and five studies did not mention the type of needle used for the irrigation—none of the studies mentioned the blinding of the evaluator (Table 6).

The authors of this review take the standpoint that all the study details need to be mentioned and focused on keeping the risk of bias low. Furthermore, the authors of this review believe that the experiments were performed according to a standardized protocol but might not have reported the intricate details since these are *in vitro* studies.

## 4. Discussion

It is well documented that primary endodontic infections are dominated by obligate anaerobic microorganisms detached from the root canal system compared to facultative bacteria [20]. It can be substantiated by the ability of flora to colonize predominantly into the main canal and least colonization into the complex areas of the root canal system [21]. Once established within the root canal system, these bacteria are difficult to eradicate as they can survive in extreme conditions. Attaining three-dimensional disinfection becomes more complex, predominantly when the microorganisms get colonized into intercanal communication, fins, cul-de-sac, and isthmus [22]. Mechanical enlargement of the root canal alone cannot eradicate the microorganisms from the root canal system, which eventually emphasizes disinfection.

### 4.1. Call for Action

Considering the disinfection for the root canal system, various factors need to be taken into account. It depends on various factors such as endodontic microbiota, access cavity design, canal preparation technique, property of the agents used for canal disinfection, the volume and contact time of the disinfectant, and the choice of the irrigant needle used for disinfection. Assessing the antimicrobial efficiency, eight out of nine studies showed a low risk of bias in reporting the outcome data [5, 11, 16, 18].

Studies have shown that nearly 70% of bacterial species invade the dentinal tubule, predominantly in apical periodontitis, discussing the microflora. The notable point was that more bacterial invasion was dominant in the coronal and middle third of the root canal system [23]. With the reduction in the oxygen potential in the apical third area, it creates a favourable environment for these organisms to establish and further colonize [24]. The virulence factors produced by these bacteria play a critical determinant role in the sustainability of these organisms [25]. Therefore, considering the abovementioned factors, the endodontic treatment should be carried out with the rationale of attaining the eradication of microbial species, although complete elimination is not possible [26].

Access cavity design plays an essential role in the disinfection of the root canal system. The cavity design should aim to provide straight-line access, facilitating the ease of instrumentation and allowing the irrigant flow to reach the working length [27]. The critical factor that plays a paramount role in conservative endodontic access is the incomplete elimination of necrotic tissue remnants. In such instances, the absolute eradication of microorganisms is absurd [28]. The previous study reported that even when a traditional endodontic access cavity was performed, the remaining pulp tissue was inevitable in the isthmus region [29]. Neelakantan et al. reported that complete debridement of the root canal was not achievable with contracted endodontic access as the entire pulp horn was not included in the cavity design, and deroofing of the pulp chamber was not performed [30].

Preparation of the root canal system is another crucial confounding factor. Preparation of the canal should be directed toward optimal in order to facilitate maximum disinfection [31]. Studies in literature have reported a larger preparation size with increased taper shown to exhibit better disinfection when compared to a lesser preparation size [32]. Maintaining the apical terminus diameter is a prime concern as the preparation taper and diameter of the apical terminus are interconnected [29]. It is noteworthy to mention that emphasize should be laid on circumventing overzealous preparation regarding respect for the canal morphology. Comparing the hand and rotary instruments, more uninstrumented areas were evident with hand instruments than with the rotary instruments for canal preparation [33, 34]. Apart from this, the instruments used for the canal preparation also play an essential role in accumulating dentinal mud into the apical ramification and lateral canal. It generally scraps more dentin when used with radial land and gets plugged into the isthmus, fins, and lateral canal. Therefore, the choice of canal preparation instruments should be considered as a contributing factor in attaining disinfection.

Over the years, the widely used agents for the disinfection of the root canal include sodium hypochlorite as the primary root canal irrigant and EDTA as a chelating agent. Sodium hypochlorite is a nonspecific proteolytic agent possessing antibacterial properties and dissolves the remnant pulp tissue [35]. Apart from this, it causes the dissolution of organic components of dentin. In an attempt to achieve the primary goal of eliminating the microorganisms, a higher concentration of sodium hypochlorite is used which can effectively eliminate microbes and indirectly affect the structural aspect of the root canal dentin. Due to the proteolytic action of NaOCl at a higher concentration, it promotes more removal of type 1 collagen, thus declining the tooth's strength when the contact time was more than 1 hour [36]. It is also evident that the concentration of the NaOCl influences the dissolving property. Periodic replenishment of NaOCl during instrumentation also plays a significant role in maintaining the concentration throughout the treatment procedure.

The contact time of the irrigant and its volume can be increased by lowering the concentration of NaOCl so as to reduce the cytotoxic effects. However, the studies have shown that a contact time of not less than 40 minutes with 5.25% NaOCl showed to be more effective when compared to lesser concentration for the same period with frequent replenishment [20]. The authors strongly make a standpoint that studies must adhere to the standardized irrigation regimen when they comparatively evaluate the antibacterial activity of NaOCl with herbal agents. None of the studies included in the systematic review maintained the contact time of 40 minutes for NaOCl. They concern herbal agents; there is no randomized clinical trial evidence to recommend contact time. There is no clear-cut recommendation exists regarding the volume of the irrigant. However, Zehnder suggested a beneficial role of copious amount of hypochlorite [20, 37].

It is a well-known fact that the needle's design and its insertion depth play a prime role in flow rate, which in turn influences the disinfection of the root canal system [38]. A 30-gauge side-vented needle allows the operator to place it up to 1 mm short of working length without binding. It also facilitates the ease of back and forth movement in the canal in order to reduce the exerted apical pressure and prevent the vapor lock effect. Discussing fluid dynamics, another aspect that needs to be taken into consideration is the flow rate of the irrigant. The effective disinfection of the root canal is related to the flow rate of the irrigant. The confounding factor directly related to the flow rate depends on the intrabarrel pressure, operator fatigue, thumb control of the operator, and gender of the operator [39].

### 4.2. Quantitative Review

The present systematic review discussed the studies that compared hypochlorite with herbal agents on antimicrobial efficiency. Efforts are going on in the field of research to minimize the antimicrobial resistance developed using conventional chemical agents by investigating the efficiency of herbal agents to replace the existing gold-standard agents. The authors of this review put forth a standpoint on whether these agents can become an alternative for canal disinfection.

Studies' included in the current systematic review used various herbal agents as a root canal irrigant. They included herbal agents such as oregano extract, triphala, green tea polyphenol, *Morinda citrifolia*, neem, carvacrol, tulsi, *Ocimum sanctum*, *Cinnamomum zeylanicum*, *Syzygium aromaticum*, and *Zataria multiflora*.

Discussing the individual agents, among all the herbal agents used, triphala, green tea polyphenol, and *Morinda citrifolia* were used in 4 studies [11, 12, 14, 17]. From the abovementioned herbal agents, triphala was found to be the most effective antimicrobial agent, followed by green tea polyphenol, and *Morinda citrifolia* was found to be the least effective. The included studies which used oregano extract and neem showed conflicting results. Since different herbal agents were used in previous studies, there is no homogeneity of the variables in the included articles. Hence, it is impossible to conclude with a single herbal agent as an effective irrigant for canal disinfection. However, its antimicrobial activity was the least when compared with hypochlorite.

Previous studies have used different concentrations of hypochlorite. This variation could be a significant confounding factor in the outcome results, which should also be taken into consideration. Therefore, it is not possible to conclude that one herbal agent is a better alternative irrigant for smear layer removal as there are variables in the included studies.

### 4.3. Qualitative Review

Meta-analysis could not be performed due to the heterogeneity of the included articles and variation in the included studies.

### 4.4. Inference

The authors of this review infer that the herbal agents cannot be a substitute for hypochlorite concerning the antimicrobial properties. All the included studies in this review showed inferior antimicrobial efficacy of the herbal agents (triphala, green tea extract, *Morinda citrifolia*, neem, carvacrol, tulsi, *Ocimum sanctum*, *Cinnamomum zeylanicum*, and Syzygium aromaticum). Only oregano oil and *Zataria multiflora* showed similar antimicrobial efficiency as sodium hypochlorite [5, 13].

### 4.5. Future Inference

The present systematic review was at the *in vitro* level; therefore, the results cannot be translated into the exact clinical conditions. Studies should concentrate on the concentration, type, volume, and contact time of these herbal agents such that they can either be used as an alternative to minimize antimicrobial resistance or as an adjunct to the already existing conventional agents. Furthermore, future studies can concentrate on whether there is a difference in the efficiency of these herbal when used freshly after the extract preparation compared to stored extract. More clinical trials should be performed to uncover any precipitate formation using these agents when used in combination and to assess if any discoloration is observed.

## 5. Conclusion

Within the limitations of the study, taking into consideration various factors, herbal agents showed less efficiency than different concentrations of sodium hypochlorite regarding the antimicrobial property. The authors of this systematic review put forth the standpoint that though results do not translate the clinical scenario, they cannot furnish definitive evidence in reporting the outcome. However, future studies can focus on the use of these herbal agents in attaining optimal disinfection.

## Figures and Tables

**Table 1:** Included article.

S. No	Author	Title of included article
1	Ok et al. [5]	Antibacterial and smear layer removal capabilities of oregano extract solution
2	Pujar et al. [11]	Comparison of antimicrobial efficacy of triphala, green tea polyphenols (GTP), and 3% of sodium hypochlorite on *Enterococcus faecalis* biofilms formed on tooth substrate: in vitro
3	Choudhary et al. [12]	Exploring the role of *Morinda citrifolia* and triphala juice in root canal irrigation: an ex vivo study
4	Sedigh-Shams et al. [13]	In vitro comparison of antimicrobial effects of sodium hypochlorite solution and *Zataria multiflora* essential oil as irrigants in root canals contaminated with *Candida albicans*
5	Rosaline et al. [14]	Influence of various herbal irrigants as a final rinse on the adherence of *Enterococcus faecalis* by fluorescence confocal laser scanning microscope
6	Gupta-Wadhwa et al. [15]	Comparative evaluation of the antimicrobial efficacy of three herbal irrigants in reducing intracanal *E. faecalis* populations: an in vitro study
7	Sharifian et al. [16]	Antibacterial substantivity of carvacrol and sodium hypochlorite in infected bovine root dentin
8	Divia et al. [17]	A comparative evaluation of *Morinda citrifolia*, green tea polyphenols, and triphala with 5% sodium hypochlorite as an endodontic irrigant against *Enterococcus faecalis*: an in vitro study
9	Arvind Kumar et al. [18]	Comparative evaluation of antibacterial and smear layer removal efficacy of two different herbal irrigants: an in vitro study

**Table 2:** General information of included article.

Author and year	Selection of teeth	Sample size	Herbal irrigant	Positive control (% NaOCl)	Negative control	Other irrigants
OK et al. 2015 [5]	Permanent maxillary central incisors	*N* = 180	Group 3: 1% oregano extract solution, group4 : 2% oregano extract solution, and group5: 5% oregano extract solution	Group 6: 5.25% NaOCl	Group 7: sterile saline	Group 1: 17% EDTA and group2: 2% chlorhexidine
Pujar et al. 2011 [11]	Permanent single rooted premolar	*N* = 40	Group 1: 60 mg/ml of triphala in 10% DMSO and group 2: 60 mg/ml of green tea polyphenol in 10% DMSO	Group 3: 3% NaOCl	Group 4: sterile saline	
Choudhary et al. 2018 [12]	Permanent single rooted teeth	*N* = 84	Group 1 (*N* = 16): MCJ and group2 (*N* = 16): triphala juice	Group 3: 1% NaOCl	Group 6: sterile distilled water	Group 4: 2% chlorhexidine and group 5: preservative control group
Sedigh-Shams et al. 2015 [13]	Permanent mandibular premolar	*N* = 60	Group 1: a minimum fungicidal concentration (MFC) of *Z. multiflora* EO (1 mg/ml) of 1 : 1024 and group 2: twice the MFC of *Z. multiflora*	Group 3: 1: 16 MFC of 5% NaOCl (3 mg/ml)	Group 4: distilled water	
Rosaline et al. 2013 [14]	Permanent single rooted teeth	*N* = 50	Group 3: 1.25 mg/ml of *Morinda citrifolia*, group 4 : 0.33 mg/ml of *Azadiracta indica*, and group 5 : 0.33 mg/ml of green tea polyphenol	Group 2: 5.25% NaOCl	Group 1: sterile saline	
Gupta-Wadhwa et al. 2016 [15]	Permanent maxillary and mandibular single rooted teeth	*N* = 40	Group A: 40% O. sanctum, group B: 10% S. aromaticum, and group C: 10% C. zeylanicum	Group D: 3% NaOCl	Group E: distilled water	
Sharifian et al. 2009 [16]	Bovine incisors	*N* = 120	Group 2: carvacrol 10%	Group 1: NaOCl 5.25%	Group 3: infected dentin tube	Group 4: sterile dentin tube
Divia et al. 2018 [17]	Permanent premolar teeth	*N* = 60	Group 3: *Morinda citrifolia* (64 mg/ml in 10% DMSO), group 4: triphala (64 mg/ml in 10% DMSO), and group 5: green tea polyphenols (64 mg/ml in 10% DMSO)	Group 2: 5% NaOCl	Group 1: distilled water	
Kumar et al. 2018 [18]	Maxillary central incisors	*N* = 120 antimicrobial efficacy (*N* = 60) and smear layer removal efficacy (*N* = 60)	Group IB: 25% neem extract (*N* = 20) and group IC: 25% tulsi Extract (*N* = 20)	Group IA: 3% NaOCl (*N* = 20)		

**Table 3:** Methodology assessment.

Author and year	Microbial inoculation	Root canal preparation (instruments used and size of preparation)	Irrigation protocol	Volume of irrigant	Time of irrigation	Needle used for irrigation	Irrigant activation devices used
Ok et al. 2015 [5]	*E. faecalis* (ATCC 29212) cultured in a BHI agar suspension adjusted to 1 × 10^8^ CFU	ProTaper NiTi rotary files 30.06% taper	No protocol mentioned	6 ml of each irrigant	2 min	Not mentioned in the study	Nil
Pujar et al. 2011 [11]	*E. faecalis* cultured in a BHI agar (strain not mentioned)	Step back upto 40 K file	No protocol mentioned	3 ml of each irrigant	10 mins	Not mentioned in the study	Nil
Choudhary et al. 2018 [12]	*E. faecalis* (MTCC 2729) and *C. albicans* (MTCC 1637) in a BHI agar is inoculated in 5 mL of suspension to obtain 1 : 1010 dilution	Step back upto 40 K file	During canal preparation, 3 mL of respective irrigant was used for 15 mins after enlargement, 2 mL of irrigant solution was used to rinse debris in the canals for another 5 min. Sterile normal saline (2 mL) was used as a final rinse	5 ml of each irrigant	20 mins	30-gauge needle	Nil
Sedigh-Shams et al. 2015 [13]	*C. albicans* in sabouraud dextrose agar suspensions adjusted to 1.5 × 10^8^ CFU	ProTaper NiTi rotary files 30.06% taper	During canal preparation, 10 ml of respective irrigants were used. Groups 1 and 2 were irrigated with 2 ml of sterile distilled water to remove the remaining *Z. multiflora* EO. Group 3 were irrigated with 2 ml of 4% sterile sodium thiosulfate solution to neutralize the remaining NaOCl	10 ml of each irrigant	12–14 mins	27-gauge needle	Nil
Rosaline et al. 2013 [14]	*E. faecalis* (ATCC 29212) cultured in tryptone bile X-glucuronide agar suspensions adjusted to 1 × 10^6^ cells/ml	Not mentioned in the study	All the specimens were treated with 5.25% NaOCl for 30 min followed by 5 mmol/L 17% EDTA for 5 mins. After which, the final irrigants were used	Not mentioned about the volume of final rinse	Final irrigation for 30 mins	Not mentioned in study	Nil
Gupta–Wadhwa et al. 2016 [15]	*E. faecalis* (ATCC 29212) suspensions adjusted to 1.5 × 10^8^ CFU	ProTaper NiTi rotary files 30.06% taper	Initially, 2 mL of experimental extract for 30 s; during instrumentation, the canal was irrigated with 2 mL of the tested extract. After instrumentation experimental, extract was left undisturbed for 60 s and then finally irrigated with 2 mL of 3% NaOCl followed by 5 mL of 17% EDTA for 1 min and again with 2 mL of experimental extract	20 ml used in each canal	6mins 30 s approx.	30-gauge needle	Nil
Sharifian et al. 2009 [16]	*E. faecalis* (ATCC 29212)	Specimens enlarged low-speed round burs of ISO sizes 025, 027, 029, 031, and 033	No protocol was mentioned	Not mentioned	20 mins contact time of irrigant	Not mentioned	Nil
Divia et al. 2018 [17]	*E. faecalis* (ATCC 29212)	Step back upto 50 k file	No protocol was mentioned	Not mentioned	Not mentioned	Not mentioned	Nil
Kumar et al. 2018 [18]	*E. faecalis* (strain not mentioned)	Step back up to 30K size	No protocol was mentioned	6 ml of irrigants	At the rate of 2 ml/15 seconds	25-gauge needle	Nil

**Table 4:** Methodology assessment.

Author and year	Sample collection	Culture plate	Assessment method	Statistical analysis performed
Ok et al. 2015 [5]	The sample was collected in an Eppendorf tube containing BHI broth	BHI and blood agar broth	Colony forming units	Kruskal–Wallis test and mann–Whitney *U* test
Pujar et al. 2011 [11]	The sample was scraped from the root canal	BHI broth	Colony forming units	One-way analysis of variance with post hoc tukey tests
Choudhary et al. 2018 [12]	Sterile paper points were inserted into the canal to collect samples	BHI agar plate and SDA	Colony forming units	Intragroup comparison of Friedman's two-way analysis of variance by ranks and post hoc Wilcoxon signed-rank test. Intergroup comparison of Kruskal–Wallis test
Sedigh-Shams et al. 2015 [13]	Sterile paper points were inserted into the canal to collect samples	SDA agar plate	Colony forming units	Kruskal–Wallis and Mann–Whitney tests
Rosaline et al. 2013 [14]	Dentin specimen was spread on the slide. Stained with BacLight	Confocal laser scanning microscopy	Bacteria counted by a manual digital counter	One-way ANOVA
Gupta-Wadhwa et al. 2016 [15]	Paper point was used to the collect sample	PCR BHI agar plate	Bacterial DNA isolation and detection colony forming units	Student's *t*-test, the mann–whitney test, Kruskal–Wallis test, and Dunn's multiple comparison test
Sharifian et al. 2009 [16]	Dentin chips were collected from the bur	BHI agar plate	Colony forming units	One-way analysis of variance and the post hoc test (Tukey)
Divia et al. 2018 [17]	Not mentioned in the study	Not mentioned in the study	Colony forming units	Kruskal–Wallis test and student's *t*-test
Kumar et al. 2018 [18]	Sample collected using H file	Real time quantitative PCR	Bacterial DNA isolation and detection	One-way analysis of variance with post hoc test (Tukey)

**Table 5:** The results of the assessment.

Author and year	Reduction in bacterial load	Outcome
Ok et al. 2015 [5]	Mean of the Log_10_ CFU and SD: 1% oregano extract—1.404 ± 1.803 CFU/ml and 5.25% NaOCl—2.308 ± 1.739 CFU/ml	1% oregano extract solution showed a similar result to 5.25% NaOCl
Pujar et al. 2011 [11]	Mean of the Log_10_ CFU and SD: triphala 2.3 ± 0.59 × 104 CFU/ml, green tea 3.8 ± 0.79 × 104 CFU/ml, and 3% NaOCl 0.00	Hypochlorite exhibited the maximum bacterial inhibition compared to triphala and green tea extract
Choudhary et al. 2018 [12]	Mean of the Log_10_ CFU and SD: *Morinda citrifolia* 3.51 ± 0.29, triphala 3.37 ± 0.56, and 1% NaOCl 1.43 ± 0.53	Compared to 1% NaOCl, morinda and triphala showed reduced effectiveness
Sedigh-Shams et al. 2015 [13]	% bacterial CFU reduction: *Z. multiflora* (2 times of MFC)—200,000 ± 0 5% NaOCl- 200,000 ± 0 *Z. multiflora* MFC—199,540 ± 313	Both *Z. multiflora* (2 times of MFC) and 5% NaOCl showed similar antimicrobial efficiency
Rosaline et al. 2013 [14]	% remaining bacterial adhesion to dentin: *Azadiracta indica* 9.30% and 5.25%, NaOCl 12.50%, green tea 27.30%, and *Morinda citrifolia* 44.20%	Compared to 5.25% NaOCl, *Azadiracta indica* showed reduced bacterial adherence
Gupta-Wadhwa et al. 2016 [15]	*Ocimum sanctum*— 2.65 × 10^5^*, Cinnamomum zeylanicum*—1.535 × 10^5^, *Syzygium aromaticum*—1.425 × 10^5^, and 3% NaOCl—1.402 × 10^4^	Compared to other herbal agents, 3% NaOCl showed better antimicrobial efficiency
Sharifian et al. 2009 [16]	Substantivity was assessed over a period of 28 days. CFU reduction (%) ±SD: carvacrol 99.3 ± 1.54 5.25% and NaOCl 99.98 ± 0.04	5.25% NaOCl showed significantly better results than carvacrol
Divia et al. 2018 [17]	CFU reduction ± SD: 5% NaOCl 0.67 ± 0.78, *Morinda citrifolia* 158.17 ± 19.83, triphala 15.92 ± 2.87, and green tea polyphenol 56.67 ± 7.18	NaOCl showed the maximum antibacterial effect, followed by triphala, green tea polyphenol, and least was *Morinda citrifolia*
Kumar et al. 2018 [18]	PCR mean value of antimicrobial efficiency: 3% NaOCl—34.20, neem—33.93, and tulsi—31.86	NaOCl showed the maximum antibacterial activity, followed by neem leaf extract. The least effect was seen with tulsi

**Table 6:** Risk of bias assessment.

Author and year	Randomization	Allocation concealment	Blinding	Standardized preparation	Reporting data
Ok et al. 2015 [5]	Unclear (teeth were randomly divided)	High (not mentioned)	High (not mentioned)	Low	Low
Pujar et al. 2011 [11]	Unclear (the samples were divided.)	High (not mentioned)	High (not mentioned)	High (strain not mentioned, irrigation protocol not mentioned, also the type of needle used)	Low
Choudhary et al. 2018 [12]	Low	High (not mentioned)	High (not mentioned)	Low	Low
Sedigh-Shams et al. 2015 [13]	High (not mentioned)	High (not mentioned)	High (not mentioned)	Low	Low
Rosaline et al. 2013 [14]	Unclear (teeth were randomly divided)	High (not mentioned)	High (not mentioned)	High (canal preparation not mentioned, the volume of irrigant used not mentioned, and the type of needle used not mentioned)	Low
Gupta-Wadhwa et al. 2016 [15]	Unclear (samples were randomly divided)	High (not mentioned)	High (not mentioned)	Low	Low
Sharifian et al. 2009 [16]	Unclear (samples were randomly divided)	High (not mentioned)	High (not mentioned)	High (irrigation protocol not mentioned, the volume of irrigant and type of needle used not mentioned)	Low
Divia et al. 2018 [17]	High (not mentioned)	High (not mentioned)	High (not mentioned)	High (irrigation protocol not mentioned, the volume of irrigant, time of irrigation, type of needle used, sample collection, and culture medium were not mentioned)	High
Kumar et al. 2018 [18]	High	High (not mentioned)	High (not mentioned)	Unclear (irrigation protocol not mentioned)	Low

## Data Availability

The data set used in the current study will be made available at a reasonable request.
